# 

**Published:** 1887-10-22

**Authors:** 


### NOTICE TO ADVERTISERS.

THE scale of charges for small prepaid Advertisements—Situations Wanted, Situations Vacant, etc., etc., is as follows :—

| | s. | d. |
|---|---|---|
| One insertion of three lines | 1 | 0 |
| Three ,, ,, ,, | 2 | 6 |
| Per line afterwards | 0 | 6 |

*A line averages ten words.*

All copy for Advertisements must be sent to A. P. WATT, 2, PATERNOSTER SQUARE, E.C., by first post on the Tuesday *preceding* publication.

### NOTICE TO CORRESPONDENTS.

All correspondence must be written on one side of the paper only.
We cannot undertake to return MSS. not used.
**Communications**, whether intended for insertion or for private information, must be authenticated by the names and addresses of the writers, not necessarily for publication.
**Local papers** containing reports or news paragraphs **should be marked.**
It is especially requested that early intelligence of **local events of interest**, or which it may be thought desirable to bring under notice, may be sent direct to the Editor.
Communications respecting editorial matters should be addressed to the Editor, Norfolk House, Norfolk Street, Strand, London, W.C.

### Amusements with Prizes, for all Ages.

**Rules.**—All answers must be addressed to the Prize Editor, The Hospital, Norfolk House, Norfolk Street, Strand, W.C., not later than the *first* post on Friday next. The full name and address must in all cases be given, but a *nom de plume* may be added for publication. Only one side of the paper must be written on. No one will be permitted to win the Monthly or Quarterly Prizes twice in any one year, the annual prizes being offered especially to prevent this.

### NEW PRIZE COMPETITION.

Only twelve competitors having entered, the conditions of the two special prizes have not been fulfilled. This is disappointing, and is not representative, we know, of the large number of readers who take an interest in this column. In these circumstances, we have decided to extend the time for sending in suggestions for the two special prizes of one guinea, as set forth on p. 18 of Volume III, being the number for October 1st, 1887.

It will be remembered that we offered an extra prize of one guinea to the correspondent who sends the names and addresses of the greatest number of subscribers, old or new, who will undertake to enter for the prizes offered to men and women during the ensuing year, and a further prize of one guinea for the best suggestion for increasing the interest in these competitions. These prizes are subject to the condition that at least twenty competitors enter for each of them, and that the lists and suggestions reach us not later than Friday, the 28th inst. All answers to be addressed to The Editor, The Lodge, Porchester Square, London, W.

In the meantime we defer the publication of the names of competitors until the 5th prox.

### FOR MEN AND WOMEN.

The results of those competitions which ended on the 15th inst. will be announced next week.

### Answers.

No. 23. Double Acrostic.

D enia L

I cen I

C el T

T im E

I ll-tempe R

O dess A

N ugge T

A die U

R ive R

Y or E

Correct answers have been received from Condorcet, Mater. Aralc, Brownie.

No. 24. Anagram.

Persia, praise, aspire.

Correct answers have been received from K. M., Condorcet, Brisbane, Ellice, Gretta, Mater, Aralc, Brownie.

### THE CHILDREN'S CORNER.

PRIZES.

FOR MOST CORRECT ANSWERS TO PUZZLES.
One Pound per Month.

FOR BEST LITTLE LETTERS.
Ten Shillings per Quarter.

A PRIZE OF THREE GUINEAS
to the Competitor who shall have sent in the largest number of correct or best answers during the twelve months.

### Puzzles—Fourth Week.

No. 12. Arithmetical Puzzle. One-third of the boys in my school are English, one-fourth French, one-fifth German, one-sixth Canadian, besides six others representing different nations. How many are there in all?

No. 13. Who can decipher the following Historical Puzzle?

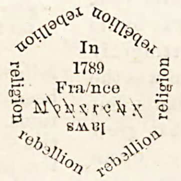

No. 14. Conundrum. What goes over the water and under the water without touching it?

* * * * No. 15. Square Word. 1. Food. 2. A girl's name. 3. A mineral. 4. Subdued.

### Letters—Fourth Week.

Next week tell me all you can about *Windsor Castle.*

### Results of Second Week—Puzzles.

No. 5. Buried Rivers. These are the Ouse, Isis, Oder.

No. 6. Pyramid Puzzle. I am very much pleased with the way in which the answers to the Pyramid Puzzle have been sent in. They were all distinguished by neatness, and in two cases, Paddy and Skye, have produced quite an artistic effect by painting the various pieces.

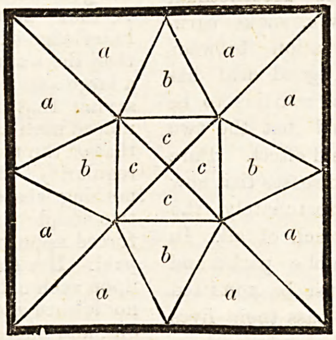

No. 7. Biographical Charade. The earl who lost his life in a rebellion against Edward II was Lancaster.

No. 8. Charade. Wheelbarrow.

RESULTS.

*All right*—A. C. K., Alsie, Mount Edgcumbe, Scrooge, Jerusalem, Toby, G. S. P., Judy, Joe, Paddy, Tim, Pepita, Skye, Ossian; *Three right* — Faith, Fern, Gem, Punch, Princess Ida; *Two right*—Poodle, Jolly Sailor, A. G. S. McCulloch, The Eda.

Poets and their Poems.

### Letters.

The letter-subject for last week has proved to be a favourite one with all my little friends. Tennyson, Longfellow, Scott, Macaulay, Hood, Mrs. Hemans, Adelaide Proctor, have all received an equal amount of praise. Gem takes Macaulay's poem *Horatius* as his favourite, and writes a nice little letter on the subject. Skye relates the story about Sir Walter Scott, called *The Cat out of the Bag.* Mount Edgcumbe and Twaddle admire the power possessed by Hood of depicting alike tragedy and comedy, giving examples of both styles. Few children have as yet studied Shakespeare, I am glad that many are looking forward to this as a great pleasure in the future. The Eda, a loyal Scotchman, sends the following interesting account of Sir Walter Scott:—

I do not know much about poets and their poems, as I have hardly read any; but the poet I like the best is Sir Walter Scott, the "Wizard of the North" he is called; I suppose because he wrote so beautifully on the lochs of Scotland, and the legends or tales of the Highlands, that he made it loved and known all over the world, instead of an almost unknown and savage country. He was born in Edinburgh, and his house is pointed out in Castle Street; and I believe Pet Margery's house, where she lived during her little life, is not far off. She used to go and see him, and he was very fond of her. She was only seven or eight when she died. His first poem is my favourite, *The Lay of the Last Minstrel* The part I like very much is about the love of country, where he begins:

"O Caledonia, stern and wild,"

for I think there is no place like dear, dear old Scotland, with its beautiful purple heather and its bracken fading into gold as it dies, and the red rowan-berries and brown Hieland burns, and the mist hanging purple over the far-away hills, and I wish I had been Sir Walter Scott, to be able to write about them myself.

Three marks each:—Twaddle, The Eda, Princess Ida, Skye. Two marks each—Mount Edgcumbe, Gem. One mark each—Ossian, Fern.

### Notices to Correspondents.

N.B.—All letters *must* reach Norfolk House *not later than first post on Fridays*, otherwise they cannot be credited.

Twaddle.—The Quarterly Letter Competition ends on December 24th. Coupon will be found on page iv.

Gem.—Please write on one side of the paper only.

